# Self-control and problematic mobile phone use in Chinese college students: the mediating role of mobile phone use patterns

**DOI:** 10.1186/s12888-016-1131-z

**Published:** 2016-11-22

**Authors:** Zhaocai Jiang, Xiuxin Zhao

**Affiliations:** Department of Psychology, School of Educational Science, Ludong University, Hongqi Middle Road 186, Zhifu District, Yantai, 264025 China

**Keywords:** Problematic mobile phone use, Self-control, Use patterns, College students

## Abstract

**Background:**

With the popularity of mobile phones, problematic mobile phone use is getting increasing attention in recent years. Although self-control was found to be a critical predictor of problematic mobile phone use, no study has ever explored the association between self-control and mobile phone use patterns as well as the possible pathway how self-control affects problematic mobile phone use.

**Methods:**

Four hundred sixty-eight college students were randomly selected in this study. Data were collected using the Problematic Mobile Phone Use Scale, the Self-Control Scale, and the Mobile Phone Use Pattern Questionnaire. Statistical tests were conducted to identify the potential role of mobile phone use patterns in the association between self-control and problematic mobile phone use.

**Results:**

In this sample, female students displayed significant higher mobile phone dependence than males. Self-control was negatively correlated with interpersonal, transaction and entertainment mobile phone use patterns, but positively correlated with information seeking use pattern. Self-control could predict problematic mobile phone use directly and indirectly via interpersonal and transaction patterns.

**Conclusions:**

Our research provided additional evidence for the negative association between self-control and problematic mobile phone use. Moreover, interpersonal and transaction use patterns played a mediating role in this link.

**Electronic supplementary material:**

The online version of this article (doi:10.1186/s12888-016-1131-z) contains supplementary material, which is available to authorized users.

## Background

Over the past decade, mobile phones are used more often than any other communication tool for Chinese people, especially young adults. By the end of 2014, the number of mobile phone users in China has reached 1.27 billion, and young adults (age 18–22) are the largest and fastest-growing group using mobile phones. The popularity of smart phones has brought more convenience for young people and increased their chances for establishing and maintaining social circle [1, 2]. Nevertheless, the disadvantages of mobile phone overuse are gradually prominent and have also been demonstrated in many studies. For instance, high frequency of mobile phone use was associated with sleep disturbance and symptoms of depression [3]. Excessive mobile phone use had a negative influence on face-to-face communication [4] and impaired social relationships were observed in problematic mobile phone users [5, 6]. Additionally, O’Connor and his colleagues found making phone calls or sending messages while driving has become one of the main causes of traffic accident in adolescents [7]. Thus, problematic mobile phone use characterized by physiological and psychological discomfort including withdrawal symptoms, social comfort as well as mood changes [8], has gained more and more attention in the past few years.

Several individual characteristics have been found to associate with problematic mobile phone use, such as extraversion [9, 10], self-esteem [11, 12] and emotional intelligence [13, 14]. Self-control is considered as another critical psychological predictor of problematic mobile phone use [15, 16]. It pertains to an individual’s capacity to resist inner desires so that he or she can achieve a more optimal outcome [17]. Previous studies have demonstrated impaired self-control and rash impulsivity are associated with compulsive buying [18], binge eating and drinking [19], pathological gambling [20] as well as internet addiction [21]. With respect to problematic mobile phone use, Khang found that compared to self-esteem and self-efficacy, self-control is the most significant predictor of pathological mobile phone use [15, 16]. Since impulsivity is an important potential contributory factor to self-control [22], several studies have shown the vital role of impulsivity in mobile phone overuse [23]. Billieux and his colleagues measured the sub-dimensions of impulsiveness and found the urgency dimension was the strongest predictor of mobile phone abuse [24]. Despite the close relationship, researchers have questioned the direct association between self-control and excessive mobile phone use [16], therefore the possible pathway how self-control affects problematic mobile phone use requires further exploration.

As for the relationship between problematic mobile phone use and use patterns, previous studies have found information-seeking pattern of users mostly using mobile phones for news-seeking and Web-surfing are not easier to pathologically rely on mobile phones than the amusement type of users mainly passing time or playing video games via phones [25, 26]. The reason may be that in comparision with entertainment functions, searching information is generally driven by certain instrumental purpose (such as learning) and brings limited pleasure [26]. Since the mobile phone is predominantly utilized for communication with others, many studies have found the social aspect of motive was particularly associated with problematic mobile phone use. For example, the interpersonal communication use motivation could predict problematic mobile phone use significantly [27]. Khang examined the roles of information-seeking, social relationship, pastime and self-presence use motives in problematic Internet, video game and mobile phone use respectively, social relationship was the only motive found to be particularly associated with problematic mobile phone use [16]. Furthermore, individuals expecting to get attention or social support via the phone tend to more emotionally involve in mobile phones, which eventually leads to pathological use [28].

On the other hand, previous studies have demonstrated personality traits could predict the type of mobile phone use that people engaged in. Adolescents scored higher on sensation seeking tend to enjoy the entertainment functions of sending and receiving pictures, playing electronic games, and downloading ring tones on their mobile phones [28]. Extraversion has been shown to associate with social communication aspect of mobile phone use and could predict the number of people called on a regular basis [9]. Butt examined 112 mobile phone owners and found extraverts spent more time calling, changing ring tone or wallpaper, implying the use of the mobile phone as a means of stimulation [12].

In terms of functionality, mobile phone is gradually completing many of the same tasks as an Internet connected computer. Specifically, with the rapid development of e-commerce in China in recent years, online transaction using mobile phone (such as online shopping, payment, transfer, etc.) has been widely accepted and become an indispensable part of college students’ daily life. Along with the continuous upgrading of mobile phone functions, research on use patterns should accordingly be updated [25]. Interpersonal, entertainment and information seeking use patterns have been extensively explored in the field of problematic mobile phone use, yet few studies attempt to evaluate the online transaction use pattern and its potential role in excessive mobile phone use. Additionally, although researchers have found the close link between self-control and problematic mobile phone use, to our knowledge, very little research has explored the association between self-control and mobile phone use pattern as well as the possible pathway how self-control affects problematic mobile phone use. Therefore, the present research focused on Chinese undergraduate students and aimed to test the following hypotheses: (1) self-control, use patterns and problematic mobile phone use are tightly associated. Self-control might positively correlate with interpersonal and entertainment use patterns, whereas negatively with information-seeking use pattern; (2) the new-emerging use pattern of online transaction can predict problematic mobile phone use significantly, while the predictive effect of some initial use patterns might fall; (3) mobile phone use pattern might play a mediating role in the relationship between self-control and problematic mobile phone use.

## Methods

### Participants

The sample consisted of 510 undergraduate students from three universities in Yantai, China. All students possess mobile phones and use it to browse the Internet as their daily activity. All subjects gave their written informed consent for inclusion before they participated in the study. The study was conducted in accordance with the Declaration of Helsinki and approved by the Institutional Review Board, sponsored by the China Association for Science and Technology and the Ministry of Health of the People’s Republic of China. A 2-stage random sampling was employed. First, out of the five universities in Yantai, three were selected at random. Second, random samples were invited through campus advertisement with the purpose of this study. Questionnaires were administered to the participants in a classroom setting during the academic years 2014–2015 by a team of trained graduate students. Before the investigation, it was emphasized that questionnaires included no identification to ensure confidentiality. The questionnaires took approximately 15 min to complete. The test administrator was present while the respondents completed the questionnaire. Of the questionnaires returned, 1 was blank and 41 were not completed. Thus, 42 invalid questionnaires had to be excluded, and the final sample consisted of 468 participants (211 males and 257 females). All students ranged in age from 18 to 24 years (M ± SD = 20.71 ± 1.47). Participants in each subgroup include: Freshmen (*n* = 104, 22.2%), Sophomores (*n* = 136, 29.1%), Juniors (*n* = 123, 26.3%), and Seniors (*n* = 105, 22.4%); Major in liberal arts (*n* = 247, 52.8%), Major in science (*n* = 221, 47.2%); From cities (*n* = 186, 39.7%), From rural areas (*n* = 282, 60.3%); Only child (*n* = 183, 39.1%), None-only child (*n* = 285, 60.9%).

### Measures

*Problematic Mobile Phone Use Scale (PMPUS)* The PMPUS is a 16-item 5 point-Likert scale developed based on Young’s [29] Internet addiction scale [8]. 1 = strongly disagree, 5 = strongly agree. It consists of four subscales: (1) withdrawal symptoms (6 items, such as “I feel lost when I do not have my mobile phone with me”); (2) salience (4 items, such as “I am obsessed with my mobile phone”); (3) social comfort (3 items, such as “I prefer to communicate by phone rather than by face-to-face talk”); (4) mood changes (3 items, such as “I feel anxious if I have not checked for messages or switched on my mobile phone for some time”). Higher score on this measure indicates greater level of mobile phone abuse. Both exploratory and confirmatory factor analyses supported the construct validity of the four subscales. It also has strong internal consistency (Chronbach’s α = 0.83) and good test–retest reliability (*r* = 0.91) [8]. Additional study proved the scale performed well with undergraduate students [30]. In this study, α = 0.87 for PMPUS, 0.71–0.77 for the four subscales.

*Self-Control Scale (SCS)* We adopted SCS based on Tangney’s Self-Control Scale [17, 31]. 19 items were preserved in view of cultural difference and reliability [31]. It measures five domains of self-control: controlling impulses (6 items, such as “I am too prone to lose my temper”), keeping healthy habits (3 items, such as “I am lazy”), resisting temptation (4 items, such as “I can resist the temptation”), focusing on work (3 items, such as “I can’t concentrate”) and controlling entertainment (3 items, such as “I do something that will give me pleasure but do harm to myself”). Participants rated how typical each statement is for them from “1 = strongly disagree” to “5 = strongly agree”. Items were reversed scored as necessary. A higher score indicated higher level of self-control. The SCS has strong internal consistency (α = 0.86) and good test–retest reliability (*r* = 0.89), proven to be a valid measure of self-control in undergraduate students [31]. In our study, α = 0.84 for the SCS, 0.72–0.78 for the five subscales.

*Mobile Phone Use Patterns Questionnaire (MPUPQ)* 15 college students were interviewed individually concerning on their daily use pattern of mobile phone. The MPUPQ was a self-compiled 5 point-Likert questionnaire taking into account of interviews with college students and previous studies [27] (see Additional file 1: Appendix 1). 1 = never, 5 = always. It consists of four dimensions: (1) interpersonal (5 items, such as “Online chatting using QQ, WeChat, etc.”); (2) entertainment (5 items, such as “Download or play games”); (3) transaction (3 items, such as “Mobile online shopping”); (4) information seeking (4 items, such as “Search or read information about learning”). Higher score in the dimension suggests greater level of use pattern. In this study, α = 0.81 for MPUPQ, 0.71–0.78 for the four subscales. Confirmatory factor analysis showed that χ^2^/df = 3.01, RMSEA = 0.05, GFI = 0.92, CFI = 0.93, NFI = 0.95, TLI = 0.94, IFI = 0.91. Thus, the questionnaire has good reliability and validity.

### Statistical analysis

The SPSS and Amos were used for data analysis. The level of significance was set at 0.05. Descriptive statistics were used to summarize and organize the data. T-tests were run to check for possible gender difference in self-control and mobile phone use behavior as well as mobile phone use difference between high and low self-control groups. Additionally, mobile phone use differences between majors/urban or rural sources/family structures were also explored by T-tests. ANOVA analysis was conducted to test the effect of grade on excessive mobile phone use. Pearson correlation coefficients were calculated to identify the possible relations between variables. In order to identify the variance of problematic mobile phone use explained by gender, self-control and mobile phone use pattern, a stepwise hierarchical regression analysis was conducted. Gender was introduced first, followed sequentially by self-control and mobile phone use pattern. For each variable, the increment of R^2^ (indicator of the contribution of each variable) is presented. Using the Amos software, structural equation modeling (SEM) with maximum likelihood estimation was used to test the mediating role of mobile phone use pattern. The model fit indices included χ^2^/df, RMSEA, GFI, CFI, NFI, TLI and IFI.

## Results

As shown in Table 1, female students displayed significantly higher PMPUS scores than male students (*t* =−3.81, *p* < 0.001, difference between means = 3.65). Yet, no significant difference was found between majors/grades/urban or rural sources/family structures. Further analysis demonstrated that compared to males, females represented higher problematic mobile phone use in all four dimensions as well as higher scores in interpersonal, entertainment and transaction use patterns (see Table 2). However, there is no significant difference in information seeking use pattern and self-control between genders.

**Table 1:** Difference of PMPUS score for genders, grades, majors, sources and family structures

Subject variables	*M ± SD*	t/F
Male	39.43 ± 10.17	
Female	43.08 ± 9.66	*t* =−3.81^***^
Liberal arts	42.56 ± 9.99	
Science	41.30 ± 9.95	*t* = 1.36
City	42.74 ± 10.80	
Rural	41.23 ± 9.37	*t* = 1.60
Only-child	41.44 ± 11.18	
None-only child	42.08 ± 9.14	*t* =−0.68
Freshman	42.55 ± 10.09	
Sophomore	42.51 ± 9.53	
Junior	41.46 ± 10.28	
Senior	41.37 ± 9.08	*F* = 2.27

Note: ^***^ *p* < 0.001

**Table 2:** Gender difference in PMPUS score, use patterns and self-control

Variables	Male *M ± SD*	Female *M ± SD*	t
Withdrawal symptoms	15.92 ± 4.72	17.86 ± 4.20	−4.38^***^
Salience	9.44 ± 2.98	10.07 ± 2.94	−2.20^*^
Social comfort	6.88 ± 2.41	7.50 ± 2.38	−2.62^**^
Mood changes	7.19 ± 2.55	7.66 ± 2.23	−2.06^*^
Interpersonal	17.48 ± 3.66	18.76 ± 2.84	−3.88^***^
Entertainment	15.01 ± 3.34	16.30 ± 2.92	−4.34^***^
Transaction	9.40 ± 2.86	10.20 ± 2.32	−3.08^**^
Information	12.63 ± 2.69	12.92 ± 2.22	−1.17
Self-control	60.01 ± 9.56	59.95 ± 9.93	0.06

Note: ^*^ *p* < 0.05; ^**^ *p* < 0.01; ^***^ *p* < 0.001

Table 3 shows the correlation coefficients of the problematic mobile phone use, use patterns and self-control. As indicated, self-control was negatively correlated with PMPUS score as well as interpersonal, entertainment and transaction use patterns (*r* =−0.40 for PMPUS, *r* =−0.14–−0.18 for use patterns; *p* < 0.01 for all), while positively correlated with information seeking pattern (*r* = 0.09, *p* < 0.05). Additionally, the level of problematic mobile phone use positively correlated with use patterns of interpersonal, entertainment and transaction (*r* = 0.17–0.27, *p* < 0.01 for all), whereas not significant correlated with information seeking use pattern (*r* = 0.03, *p* > 0.05).

**Table 3:** Pearson correlations between PMPUS, use pattern and self-control

Variables	1.PMPUS	2.Interpersonal	3.Entertainment	4.Transaction	5.Information	6.Self-control
1	—					
2	0.26^**^	—				
3	0.17^**^	0.38^**^	—			
4	0.27^**^	0.48^**^	0.43^**^	—		
5	0.03	0.43^**^	0.40^**^	0.41^**^	—	
6	−0.40^**^	−0.18^**^	−0.17^**^	−0.14^**^	0.09^*^	—

Note: ^*^ *p* < 0.05; ^**^ *p* < 0.01

To assess whether mobile phone overuse and use patterns distinguished between students displaying different levels of self-control, participants were categorized into three groups with the first 27% as high self-control group (SCS score > 65, *n* = 84) and the last 27% as low self-control group (SCS score < 55, *n* = 78). As illustrated in Table 4, the low self-control group scored significantly higher than the high self-control group on PMPUS (*t* = 7.71, *p* < 0.001) as well as use patterns of interpersonal (*t* = 2.73, *p* < 0.01), entertainment (*t* = 2.65, *p* < 0.01) and transaction (*t* = 2.56, *p* < 0.05), whereas marginally significant less on information seeking pattern (*t* =−1.78,*p* = 0.07).

**Table 4:** Variance for PMPUS and use patterns between low and high self-control groups

Variables	Low self-control *M ± SD*	High self-control *M ± SD*	t
PMPUS	46.02 ± 10.21	36.70 ± 8.62	7.71^***^
Interpersonal	19.51 ± 3.37	18.23 ± 3.22	2.73^**^
Entertainment	16.60 ± 3.11	15.60 ± 2.77	2.65^**^
Transaction	10.51 ± 2.64	9.68 ± 2.41	2.56^*^
Information	12.78 ± 2.24	13.30 ± 2.30	−1.78

Note: ^*^ *p* < 0.05; ^**^ *p* < 0.01; ^***^ *p* < 0.001

Regressing PMPUS score on gender, self-control and mobile phone use patterns are shown in Table 5. Information seeking use pattern is not included for no significant correlation with PMPUS was observed. In step 1, gender could significantly predict problematic mobile phone use (β = 0.17, *p* < 0.001), accounting for 3% of the variance of PMPUS. In step 2, self-control was the negative predictor of PMPUS (β =−0.40, *p* < 0.001), accounting for an additional 16%. In step 3, the predictive effects of gender and self-control were still significant, with use patterns accounting for 5% independently. Moreover, use patterns of interpersonal and transaction could positively predict PMPUS (β = 0.15, *p* < 0.01 for interpersonal; β = 0.14, *p* <0.01 for transaction), while the predictive effect of entertainment was not significant (β =−0.04, *p* > 0.05). All these variables jointly explained 24% of the variance of PMPUS.

**Table 5:** Gender, self-control and use patterns as predictors of PMPUS

Predictors	Step 1(β)	Step 2(β)	Step 3(β)
Gender	0.17^***^	0.17^***^	0.13 ^**^
Self-control		−0.40^***^	−0.37 ^***^
Interpersonal			0.15 ^**^
Entertainment			−0.04
Transaction			0.14^**^
ΔR^2^	0.03	0.16	0.05
ΔF	14.54^***^	90.60^***^	10.65^***^

Note: ^**^ *p* < 0.01; ^***^ *p* < 0.001

**Fig. 1:**
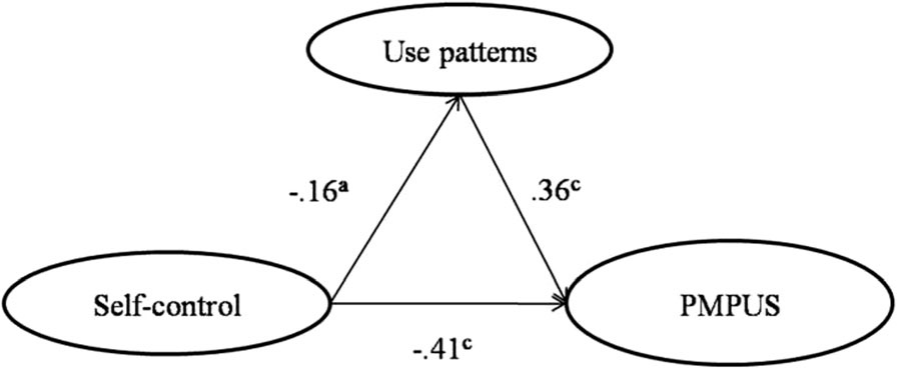
Conceptual model of the mediating effect of mobile phone use patterns. Note: ^a^ *p* < 0.05; ^c^ *p* < 0.001

The hypothesis model of this research assumes use patterns mediate the relationship between self-control and problematic mobile phone use. As shown in Fig. 1, the model fitting values are χ^2^/df = 2.26, RMSEA = 0.05, GFI = 0.97, CFI = 0.97, NFI = 0.94, TLI = 0.95, IFI = 0.97, indicating the model fit is ideal. Thus, self-control can directly predict college students’ problematic mobile phone use, and indirectly via interpersonal and transaction use patterns. The mediating effect accounted for 12.77% of the total effect.

## Discussion

The purpose of this study was to examine the association among self-control, mobile phone use patterns as well as problematic mobile phone use and to evaluate the mediating role of use patterns between self-control and problematic mobile phone use in college students. The results revealed that females exhibited higher scores in interpersonal, entertainment and transaction use patterns as well as mobile phone dependence than males. Use patterns of interpersonal, entertainment and transaction correlated positively with problematic mobile phone use, whereas negatively with self-control. Additionally, information-seeking pattern was positively correlated with self-control, while no significant correlation with mobile phone dependence. Furthermore, use patterns of interpersonal and transaction rather than entertainment and information seeking could effectively predict problematic mobile phone use. Self-control could predict problematic mobile phone use directly and indirectly via interpersonal and transaction use patterns. Our research provided additional evidence for the association between self-control and problematic mobile phone use and indicated the mediating effect of interpersonal and transaction use patterns in this link.

Consistent with previous studies [5, 13], we found compared to males, females are more likely to depend on mobile phone and gender is the significant predictor of problematic mobile phone use. Our further analysis showed females displayed higher scores in use patterns of interpersonal, entertainment and transaction than males. In accord with our results, other studies also found females tend to use mobile phones to establish and maintain social relationships, while males prefer to use it in the workplace [9, 32, 33]. Maintaining social relationships usually requires more emotional involvement than searching information. More importantly, females naturally tend to exhibit more time and emotional investment on shopping and interpersonal communication than males [34]. Additionally, females who score higher in the mobile phone dependence tend to pay more attention to their emotions and thus more easily to experience emotion disturbance caused by mobile phone overuse [13]. Therefore, we speculate that female college students are inclined to deal with more emotion-involved issues with the phone, ruminate on these emotions and thus result in heightened mobile phone dependence.

As previous studies indicated [16, 26, 27], we found interpersonal, entertainment and transaction use patterns correlated positively with problematic mobile phone use, whereas for information seeking, the correlation was not significant. Additionally, previous studies have suggested interpersonal and entertainment both effectively predicted problematic mobile phone use [25, 27]. However, when transaction was included in the present study, it replaced entertainment becoming an effective predictor of excessive mobile phone use. The 36th Statistical Report on Internet Development in China showed that mobile stock, travel reservation, online payment and online shopping became the fastest growing mobile applications from December 2014 to June 2015. College students are comparatively easy to embrace various new technologies [35] and thus fresh sense of online transaction may be one important reason of excessive use. Apart from this, late adolescents and young adults are likely to engage in activities for the purposes of establishing social relationships and developing a sense of identity [35]. Buying is considered as an identity-seeking behavior and young people tend to symbolize their identity and enhance emotional state in this way [34, 36]. Thus, online shopping with smart phone might become a convenient way for college students to seek out and express self-identity. In contrast, ways of entertainment are gradually diversified in recent years. Due to better audio-visual experience, new entertainment means (such as iPad, handheld game console, etc.) have partly replaced the recreational function of the mobile phone. Our results suggest that more importance should be attached to transaction use pattern when examining or intervening problematic mobile phone use in young adults. As time goes on, whether transaction use pattern could still effectively predict mobile phone abuse needs to be reevaluated in the future.

In agreement with previous research [15, 16, 24], we found low self-control participants are more susceptible to use their mobile phones pathologically. Self-control could predict problematic mobile phone use directly and indirectly via interpersonal and transaction use patterns. Self-control refers to the ability to forgo immediate rewards for the sake of achieving long-term goals [37, 38]. Thus, individuals displaying higher level of self-control tend to voluntarily choose activities beneficial in the long run and obtain satisfaction through engaging in or accomplishing activities. On the other hand, a low self-control individual prefers to choose activities which directly accompany reward and initially bring more joy. In fact, when faced with a reward signal, lower self-controllers tend to easily experience positive emotions and exhibit approach behavior [39]. Most of the time, interpersonal communicating and shopping both bring fresh stimulations and positive emotions. Thus, initially low self-control students are more likely to be attracted and absorbed by these functions. Then, pleasure and satisfaction benefiting from these functions will drive them to use more, which may eventually result in excessive use.

Additionally, low self-control students exhibited lower self-esteem, worse academic performance and interpersonal relationship along with unhealthier lifestyle versus students with high self-control [17, 40, 41]. Inadequate self-control tends to be highly correlated with a negative state of mind [17]. For low self-controllers, mobile phone featuring mobility and portability may become an important way to regulate their negative emotions [42]. However, the passive coping style of diverting attention only temporarily alleviates negative emotions and could not change their unfavorable situation. More importantly, the negative emotional state is susceptible to interfere with executive control [43] and thus result in compulsive mobile phone use. Therefore, in addition to the initial function selection, self-control may regulate the entire process of mobile phone use and act as the critical factor protecting students from problematic mobile phone use.

Some limitations of this study should be noted. First of all, the cross-sectional design of the present study could not confirm causal relationships between problematic mobile phone use and possible influential factors. Future longitudinal studies are needed to identify the causal relationship. Secondly, online shopping using smart phone has just begun to pop up in China in recent years. In the present study, only college students characterized by relatively high adaption to new technology were included. Thus, our results require to be tested in other samples, such as a community sample. Thirdly, in order to ensure full involvement of learning tasks, students are forbidden to use mobile phone in class in the three colleges we surveyed. It is possible that this rule may increase students’ awareness of disadvantages of excessive mobile phone use and thus lead to an underestimation of the prevalence of problematic mobile phone use in our study.

## Conclusions

The current study examined 468 college students and found self-control was negatively associated with interpersonal, transaction and entertainment mobile phone use patterns, but positively with information seeking use pattern. Use patterns of interpersonal and transaction rather than entertainment and information seeking could effectively predict problematic mobile phone use. Self-control could predict mobile phone overuse directly and indirectly via interpersonal and transaction use patterns. To better understand mobile phone abuse behavior, the fact that new–emerging transaction use pattern has become a critical predictor of problematic mobile phone use should be more emphasized. Moreover, our research indicates that helping problematic mobile phone users to rebuild self-control will be of great importance during treatment in the future.

## Additional file

Additional file 1: Appendix 1. Mobile Phone Use Patterns Questionnaire (MPUPQ) (translated from the original Chinese). (DOCX 16 kb)

## Abbreviations

ANOVA: Analysis of variance
CFI: Comparative fit index
GFI: Goodness of fit index
IFI: Incremental fit index
MPUPQ: Mobile phone use patterns questionnaire
NFI: Normed fit index
PMPUS: Problematic mobile phone use scale
RMSEA: Root mean square error of approximation
SCS: Self-control scale
SD: Standard deviation
TLI: Tucker lewis index
