# Supplementary material for: Self-control and problematic mobile phone use in Chinese college students: the mediating role of mobile phone use patterns
Source: BMC Psychiatry. 2016 Nov 22;16:416. doi: 10.1186/s12888-016-1131-z (PMC5120559; doi:10.1186/s12888-016-1131-z)
Supplement: Additional file 1: Appendix 1. — Mobile Phone Use Patterns Questionnaire (MPUPQ) (translated from the original Chinese). (DOCX 16 kb) [file 12888_2016_1131_MOESM1_ESM.docx]

How often the following functions are used when you use your mobile phone every day. Please draw “√” in the corresponding figure.

|  | 1  never | 2  rarely | 3  sometimes | 4  often | 5 always |
| --- | --- | --- | --- | --- | --- |
| Make mobile phone calls (including Internet telephony) | 1 | 2 | 3 | 4 | 5 |
| Selfie or take photos for others | 1 | 2 | 3 | 4 | 5 |
| Mobile online shopping | 1 | 2 | 3 | 4 | 5 |
| Send or receive short messages | 1 | 2 | 3 | 4 | 5 |
| Send or receive E-mails | 1 | 2 | 3 | 4 | 5 |
| Download or read cartoons, online novels, etc. | 1 | 2 | 3 | 4 | 5 |
| Search or read information about learning | 1 | 2 | 3 | 4 | 5 |
| Download or listen to music | 1 | 2 | 3 | 4 | 5 |
| Query life information (such as weather, maps, etc.) | 1 | 2 | 3 | 4 | 5 |
| Online chatting using QQ, WeChat, etc | 1 | 2 | 3 | 4 | 5 |
| Download or watch videos (such as TV shows, movies, etc) | 1 | 2 | 3 | 4 | 5 |
| Browse online news | 1 | 2 | 3 | 4 | 5 |
| Browse social networking sites (such as QQ Zone, WeChat circle of friends, etc.) | 1 | 2 | 3 | 4 | 5 |
| Mobile online reservation | 1 | 2 | 3 | 4 | 5 |
| Release/share the state or photographs on social networks | 1 | 2 | 3 | 4 | 5 |
| Mobile payment or mobile bank transfer | 1 | 2 | 3 | 4 | 5 |
| Download or play games | 1 | 2 | 3 | 4 | 5 |
